# Physical Activity and DNA Methylation in Humans

**DOI:** 10.3390/ijms222312989

**Published:** 2021-11-30

**Authors:** Witold Józef Światowy, Hanna Drzewiecka, Michalina Kliber, Maria Sąsiadek, Paweł Karpiński, Andrzej Pławski, Paweł Piotr Jagodziński

**Affiliations:** 1 Department of Biochemistry and Molecular Biology, Poznan University of Medical Sciences, 60-781 Poznan, Poland; hdrzewiecka@gmail.com (H.D.); michalina.kliber@gmail.com (M.K.); pjagodzi@ump.edu.pl (P.P.J.); 2 Department of Genetics, Wroclaw Medical University, 50-368 Wroclaw, Poland; maria.sasiadek@umed.wroc.pl (M.S.); polemiraza@poczta.fm (P.K.); 3 Institute of Human Genetics, Polish Academy of Sciences, 60-479 Poznan, Poland; andp@man.poznan.pl

**Keywords:** DNA methylation, epigenetics, exercise, physical activity

## Abstract

Physical activity is a strong stimulus influencing the overall physiology of the human body. Exercises lead to biochemical changes in various tissues and exert an impact on gene expression. Exercise-induced changes in gene expression may be mediated by epigenetic modifications, which rearrange the chromatin structure and therefore modulate its accessibility for transcription factors. One of such epigenetic mark is DNA methylation that involves an attachment of a methyl group to the fifth carbon of cytosine residue present in CG dinucleotides (CpG). DNA methylation is catalyzed by a family of DNA methyltransferases. This reversible DNA modification results in the recruitment of proteins containing methyl binding domain and further transcriptional co-repressors leading to the silencing of gene expression. The accumulation of CpG dinucleotides, referred as CpG islands, occurs at the promoter regions in a great majority of human genes. Therefore, changes in DNA methylation profile affect the transcription of multiple genes. A growing body of evidence indicates that exercise training modulates DNA methylation in muscles and adipose tissue. Some of these epigenetic markers were associated with a reduced risk of chronic diseases. This review summarizes the current knowledge about the influence of physical activity on the DNA methylation status in humans.

## 1. Introduction

**Figure 1:**
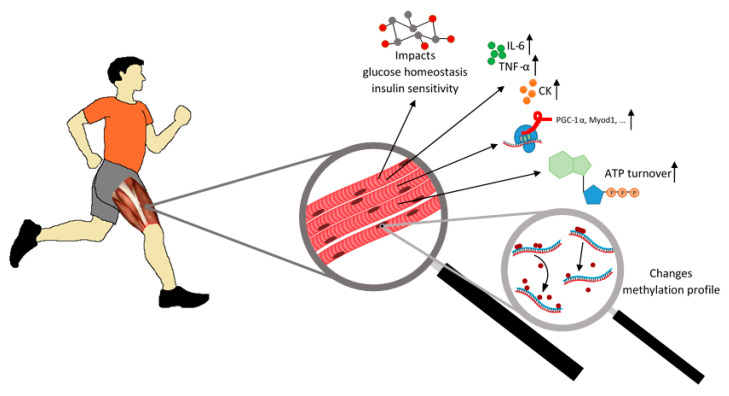
Exercise affects the metabolism of muscle cells. Exercise impacts glucose homeostasis and insulin sensitivity, enhances the release of creatine kinase (CK), and pro-inflammatory cytokines such as interleukin-6 (IL-6) and tumor necrosis factor α (TNF-α) as well as increases ATP turnover. Physical activity also induces changes in DNA methylation patterns and influences the expression of many genes in muscle tissue.

The effect of exercise on the human body is a widely studied topic. Exercise usually exerts a positive impact on the functioning and physiology of human body, improves endurance, the efficiency of the respiratory and cardiovascular system, and affects the immune system and neurophysiology [1,2]. It is well known that lack of physical activity may contribute to the development of chronic diseases and it leads to biochemical and molecular changes in particular tissues (Figure 1) [3].

**Figure 2:**
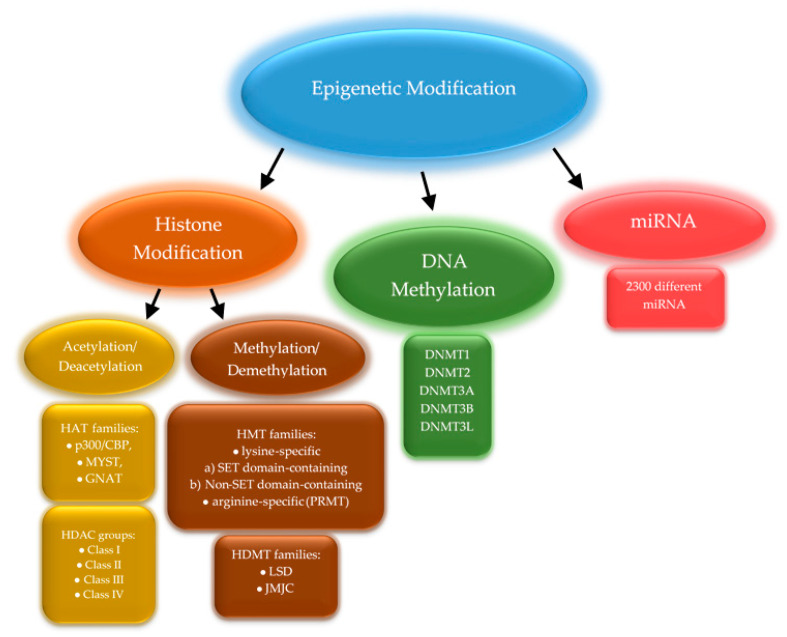
Three major epigenetic modifications. Enzymes classified into several families are responsible for the modification of histones proteins. The human DNA methyltransferase family consists of five members. The miRNAs are a diverse group of molecules with approximately 2300 different miRNAs [7].

Nowadays, scientists try to answer the question of which mechanisms underlie the multi-organ adaptation to exercise. Physical activity increases blood creatine kinase levels and triggers the secretion of pro-inflammatory cytokines, mainly interleukin-6 (IL-6), but also interleukin-1β, and tumor necrosis factor α (TNF-α) [4]. Muscle cells react differently depending on the type of exercise. Endurance training increases oxidative capacity and resistance training promotes protein synthesis, thus boosting muscle strength and inducing gains in muscle mass [5]. Exercise also influences the expression of genes related to muscle work, such as *PPARGC1A* encoding peroxisome proliferator-activated receptor gamma coactivator 1-alpha (PGC-1α)—a transcriptional coactivator that regulates genes involved in energy metabolism or *MYOD1* encoding a nuclear protein that belongs to the myogenic differentiation family of transcription factors [6]. Changes in the expression level of multiple genes result from regulatory mechanisms, including epigenetic modifications. These mainly relate to histone modifications (mostly methylation and acetylation), DNA methylation, and micro RNA (miRNA) production that impacts the translation process (Figure 2).

**Figure 3:**
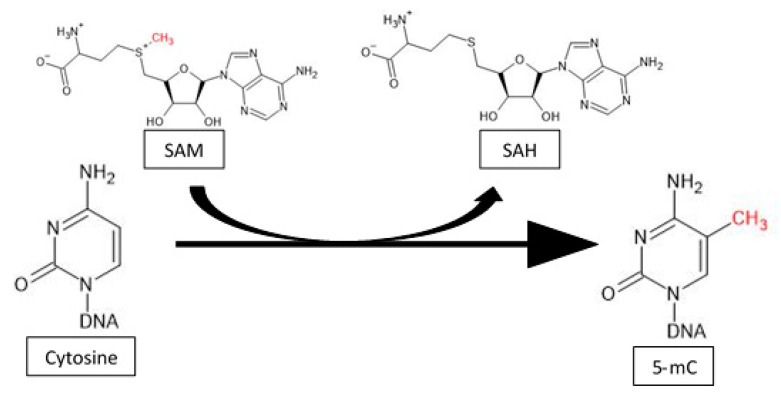
The mechanism of cytosine methylation. The mechanism of methylation is catalyzed by DNMTs. Those enzymes transfer a methyl group from SAM to the fifth carbon of a cytosine residue to form 5-methylcytosine (5-mC).

In recent years, epigenetic changes in response to exercise and their physiological consequences have been extensively studied. This review focuses on issues related to variations in DNA methylation patterns induced by physical activity. DNA methylation is catalyzed by a class of highly conserved enzymes belonging to the DNA methyltransferase family (DNMT) that comprises five members: DNMT1, DNMT2, DNMT3A, DNMT3B, and DNMT3L [8]. DNMT1 is responsible for maintaining the DNA methylation pattern onto the newly synthesized DNA strand during replication and therefore is involved in the preservation of epigenetic information. Two other enzymes—DNMT3A and DNMT3B—participate in the process of *de novo* methylation, which establishes new methylation patterns and is still the subject of extensive research. DNMT2 was shown to be a tRNA methyltransferase that can lead to the formation of tRNA fragments molecules, the role of which is still extensively studied [9,10]. DNMT3L itself does not possess any methyltransferase activity; however, it increases the activity of DNMT3A and DNMT3B, thus stimulating the process of *de novo* methylation [11]. DNA methylation involves the transfer of a methyl group to the fifth carbon of the pyrimidine ring of cytosine that precedes a guanine nucleotide (CpG sequences). The main donor of methyl groups for this reaction is S-adenosyl methionine (SAM), which is subsequently converted into S-adenosyl homocysteine (SAH) (Figure 3) [8].

An accumulation of CpG dinucleotides in genomic regions is called CpG Islands where they account for 60–70% of the nucleotides content, while outside CpG Islands these dinucleotides are rare, less than 1% [12]. CpG islands are present in 60–70% of all human gene promoters, which shows that they are essential elements in the regulation of gene expression [13]. Methylated gene promoters have a limited ability to bind transcription factors and this modification recruits methyl binding proteins, leading to chromatin condensation and gene silencing. In addition, DNA methylation is responsible for such processes as silencing the X chromosome and parental imprinting [14,15].

## 2. Global Methylation

Numerous studies indicated changes occurring in the global DNA methylation profile after exercise. Those observations confirmed that epigenetic modifications are involved in the regulation of gene expression and mediate adaptation to environmental conditions. Alterations in DNA methylation patterns are readily investigated in white blood cells, because blood samples are relatively easy to obtain. On the other hand, large adaptive changes in global DNA methylation triggered by physical activity are also expected in of skeletal muscles and in adipose tissue [16]. However, this approach demands more invasive methods for specimen collection. Currently, there are many discrepancies in various studies regarding the level of global DNA methylation in the blood connected with exercises. Different studies revealed the DNA hypomethylation or hypermethylation in peripheral blood mononuclear cells associated with physical activity and some of them depicted that exercise does not influence the level of global DNA methylation regardless of age, type of activity, current fitness level, or type of cells. An evaluation of DNA methylation levels in young trained male cyclists after acute aerobic exercise (45 min cycling with 75% maximal power output (Wmax) and 15 min time trial (TT)) indicated a significant reduction in global DNA methylation [17]. Nevertheless, other studies did not confirm such a change, but differences in CpG island methylation patterns in the promoter regions of individual genes were pinpointed. Robson-Ansley et al. did not observe any changes in DNA methylation levels in trained males after acute aerobic exercise (45 min running with 60% maximal oxygen uptake (VO_2max_) and 5 min time trial (TT)), which may indicate that these changes were at a negligible level in the respondents or the amount of hyper- and hypomethylation was at a relatively similar level [18].

However, patterns of DNA methylation changes rapidly depending on the intensity and duration of exercise. Using acceleration data, it was recognized that participants (45–75 years of age) who exercised from 26 to 30 min a day had a significantly higher level of global genomic DNA methylation compared to those individuals who trained less than 10 min a day [19]. It is well recognized that the level of DNA methylation decreases with age [20], leading to genome hypomethylation associated with genomic instability, a disturbance commonly observed during carcinogenogenesis and other metabolic diseases, such as type 2 diabetes [21,22,23,24,25]. It is proposed that exercises can extend life expectancy by maintaining methylation patterns. However, no crorrelation with an average physical activity during the day (measured by a pedometer) and DNA methylation levels in blood cells was confirmed in the elderly [26,27].

Investigations in the group of women around 70 years of age presented us an interesting juxtaposition and discrepancies. In the first study, women over 70 declaring higher physical activity were characterized by a significantly lower genome-wide DNA methylation profiles in peripheral blood mononuclear cells (PBMCs) than those declaring lower physical activity, and it was correlated to a better health status of participants [28]. Other interventional studies in the elderly confirmed these results. The period of 12 weeks of the moderate intensity and low frequency resistance training reduced the global DNA methylation level in PBMCs and exerted a beneficial effect on overall health of subjects. The subjects’ group was women with an average age of 72 years, which shows that physical activity is an important factor in improving the health of the elderly and it may be related to DNA methylation [29].

The contradictions are studies that indicate an increase in global methylation in people declaring higher physical activity. In this study, the level of global methylation was tested on the basis of LINE-1 regions, and physical activity was assessed retrospectively during childhood, adolescence period, and the last 12 months. The results, however, were not statistically significant, but showed a tendency for a positive correlation between exercise and methylation DNA in leukocytes [30]. Intervention studies in older women confirmed the earlier trend and showed a significant increase in global leukocyte methylation with resistance and aerobic exercises. Increased DNA methylation negatively correlated with the time of performing the exercise protocol, i.e., people with better physical condition, which translated into more efficient exercise, had higher DNA methylation compared to sedentary women [31].

Research depicts that changes in global methylation levels in blood cells are not a good marker of exercise. However, all studies have shown that the methylation profile was altered with exercise, and more careful research is required based on the methylation profile or pattern rather than the level of epigenetic modification itself.

Such a more detailed study reveals that this is hyper- and hypomethylation of particular regions of the genome. Studies on a group of recreationally active young men who were tested before and after eight weeks of strength training (three sets of 8–12 repetitions with 80% maximum load) showed that the genome methylation profile in leukocytes had changed. Of the 57,384 sites changed, 28,397 were methylated and 28,987 demethylated. As can be seen in this case, there is no change in the methylation level in the general sense, but the methylation profile has changed significantly [32].

Muscle cells do the work that is needed to get the body moving and exercising. Cells reorganize metabolism and adapt to exercise, and this process is under control by gene methylation in this tissue [16]. Researchers have repeatedly assessed whether and how global methylation level in skeletal muscles changes under the influence of exercise. Such changes were observed after a single acute aerobic exercise at 40% and 80% VO_2max_ in a group of young people leading a sedentary lifestyle. Global DNA methylation in this group of people has decreased, which may indicate increased gene expression in muscle cells, which coincides with increased work [6].

A similar tendency towards more demethylation processes was described in the group of older men who declared their physical activity throughout their lives. Declarations have been verified by measuring body composition and endurance. This inactive group had 39% more body fat, 13% less muscle mass, and cycling endurance was 41% lower. Changes in methylation were noted between the groups. The group of active people generally had less methylation of the gene promoters. It was found that 714 gene promoters were significantly less methylated and 31 gene promoters were significantly hypermethylated. It was noted that the regions of introns, CpG islands, and exons depicted no difference between the groups [23].

An interesting approach was presented by Lindholm et al. who studied the change in methylation on a group of sedentary volunteers who endurance trained (four 45 min sessions per week) only one leg for three months, which helped exclude environmental effects on methylation changes and many different factors that could not be excluded in most of the work. Researchers analyzed DNA methylation and gene expression in both legs in the *vastus lateralis*. Nearly 5000 sites with altered methylation in the trained leg and 4000 genes with altered expression were observed using the matrix. When examining the global methylation level, such changes were not observed either in relation to the training itself or between both legs [33].

As in the case of the analysis of blood methylome in skeletal muscle cells, there is no clear upward or downward shift in global methylation levels. Its profile changes and the total amount of methylated cytosine are the result of the hypo- and hypermethylation of the promoters of various genes, as shown by the research of Nitert et al., and the use of the global DNA methylation determination for multigene research, and not the relative amount of 5-methylcytosine [34].

Global methylation studied in adipose tissue after exercise depicts that the metabolic changes occurring in it are not only the result of biochemical changes. This change in expression is also dependent on epigenetic changes and may remain.

Extensive research on adipose tissue was carried out on non-exercising men subjected to one-hour session spinning and two one-hour sessions of aerobic per week during a 6-month endurance training. They found that the men revealed an altered methylation profile. Increased global methylation levels were observed in these studies, which may affect the metabolism of adipocytes. It was determined that 17,975 individual CpG sites located in 7663 genes were changed. Most of these sites were located in the gene bodies and intergenic regions. It was also noticed that in the case of 197 genes, there was a change in methylation and transcription, of which 58% had an inverse relationship and 97% of them were related to hypermethylation and decreased expression [35].

Epigenetic modifications also influence on other tissues and organs. For example, exercise through DNA methylation also affects the functioning of the hypothalamus. An increase in global methylation was observed in rats in this part of the brain after endurance exercise [36].

DNA methylation also plays an important role in neoplastic processes. Research indicates that global hypomethylation can cause greater genome instability and the activation of inappropriate regions of the genome. Reduced genome methylation is observed in cancer cells. This can lead to the activation of tumor suppressor genes in some types of cancer. It has also been admitted that long-term exercise through epigenetic changes reduces the risk and mortality in breast, colorectal and stomach cancer [36]. Physical exercise, by inducing hyper- and hypomethylation in the appropriate regions, contributes to the introduction and maintenance of an appropriate methylation profile, which reduces the risk of neoplastic mutations [37,38,39].

Research shows that measuring global methylation helps determine if a given environmental factor influences epigenetic modification and can help find interesting genes for more insightful analysis. Exercise is undoubtedly a powerful environmental stimulus that causes epigenetic changes every time. In muscle tissue, we have the dominance of hypomethylation processes and activation of more genes leading to an increase in cell activity. The opposite tendency is observed in fat cells, where the predominance of hypermethylation processes indicates that the functioning of the cell is limited. However, this does not mean that we do not have reverse processes in both cases. The processes of hypermethylation also occur in muscle cells, and hypomethylation in fat, aimed at the appropriate regulation of cell metabolism.

## 3. Gene Methylation

Changes in global profile methylation incline the researchers to assess metabolic pathways which play a huge role in adaptation to exercise. The starting point to analyzing DNA methylation was genes depicting a significant expression modification and including in part of relevant process in terms of the exercises performed. The type of physical activity is also important, whether single acute exercises or a training plan.

### 3.1. In Muscle

Even one-time acute exercises are able to modulate changes in the level of DNA methylation within an hour of starting the exercise. Barres et al., studying muscle cells, described hypomethylation in promoters of genes involved in mitochondrial functions and the use of fuels: Peroxisome proliferator-activated receptor gamma coactivator 1-alpha (*PPARGC1A* or *PGC-1α*), Pyruvate Dehydrogenase Kinase 4 (*PDK4*), Mitochondrial Transcription Factor 1 (*TFAM*), Peroxisome proliferator-activated receptor delta (*PPAR-δ*), citrate synthase (*CS*), and myocyte enhancer factor 2A (*MEF2A*). There was also an increase in the expression of genes tested on sedentary subjects after low- and high-intensity aerobic exercise [6]. Hypomethylation and an increase in *PGC-1α* expression was confirmed in other studies with the influence of acute aerobic exercise or electrically induced muscle exercise on DNA methylation [40,41,42].

Physical training, which is, in definition, to take place over a longer period of time, reveals that muscles adapt to exercise more strongly and that muscle cells modify the methylation profile on a larger scale. Physical training seems to reprogram the organism by using methylation processes to adapt. It is also significance that the body is able to react more strongly through DNA methylation to secondary effort, thus demonstrating epigenetic memory. This is shown by a study where two training periods with three 60 min progressive volume model sessions per week were interrupted by a rest period. The first 7-week period of resistance training resulted in 17,365 altered CpG sites, of which 9153 were hypomethylated, then after seven weeks without physical activity, there was a methylation change at 17,529 CpG sites, of which 8891 were hypomethylated. Epigenetic memory can be seen after the third period (the second a series of exercises) where 27,155 CpG sites were changed and 18,816 sites were hypomethylated. The genes that were more hypomethylated and overexpressed after two series of exercises are the RNA binding protein involved in the post-transcriptional modification of BicC Family RNA Binding Protein 1 (*BICC1*), an essential protein for the cohesion of sister chromatids after DNA replication of Stromal Antigen 1 (*STAG1*), of undefined role in muscle Glutamate Ionotropic Receptor Kainate Type Subunit 2 (*GRIK2*), and a protein involved in the proliferation and differentiation of skeletal muscle cells TNF Receptor Associated Factor 1 (*TRAF1*) [43].

*PGC-1α* has also been proven over an extended period of time. In addition, a negative stimulus was taken into account, namely, the volunteers were in bed for nine days, which prevented most of the physical activity, and then exercised for four weeks. After lying down, hypermethylation with decreased expression of *PGC-1α* was demonstrated, which may contribute to the observed transcriptional changes in genes potentially involved in the pathogenesis of insulin resistance and Type 2 Diabetes (T2D). These changes were only partially reversed after the aerobic exercise period (30 min cycling with 70% VO_2max_, 6 days/week) [44].

It also examined whether chronic exercise, as one of the risk factors for T2D, affects DNA methylation in people with and without a family history of the disease, which is another risk factor for the disease. Six-month endurance exercises with one session of one-hour spinning and two sessions of one-hour aerobic per week depicted methylation differences in 21 of 39 T2D candidate genes, of which Thyroid Adenoma Associated (*THADA*) and RNA Binding Motif Single Stranded Interacting Protein 1 (*RBMS1*) showed significant differences and 18 of them being nominal differences between groups with family history [34,45]. *RBMS1* is involved in DNA replication, transcription, and apoptosis [46]. In addition, hypomethylation and an increase in expression after endurance exercise were observed in the genes of the transcription factors *MEF2A* and Runt-Related Transcription Factor 1 (*RUNX1*), T2D-related *THADA*, NADH respiratory chain: Ubiquinone Oxidoreductase Subunit C2 (*NDUFC2*), and also Interleukin 7 (*IL-7*). There were also differences in the expression and methylation of genes involved in muscle metabolism Adiponectin Receptor 1 (*ADIPOR1*), Adiponectin Receptor 1 (*ADIPOR2*), and Bradykinin Receptor B2 (*BDKRB2*). Researchers observed hypomethylation in genes related to retinol metabolism, calcium signaling pathway, starch and sucrose metabolism, or insulin signaling pathway, and hypermethylation in genes related to purine, serine, threonine, and glycine metabolism, insulin signaling, and glycolysis and gluconeogenesis [34].

Changes in the gene *MEF2A* after endurance 3-month training (four 45 min sessions per week) are different in the group of healthy people who lead a sedentary lifestyle. Promoter hypermethylation was detected along with a decrease in the expression of this gene. These observations confirm the decrease in the expression of the *CDCH15*, *MYH3*, *TNNT2*, *RYR1,* and *SH3GLB1* genes which are upregulated by *MEF2A*. This result seems to be non-intuitive, as the authors point out, and it may be the result of dynamic changes and the observed effect is negative feedback after morphological changes or an increased representation of cell types after training [33].

The *PDK4* gene is involved in the metabolism of fuel consumption and as previously described was hypomethylated after acute aerobic exercises in diabetics. It has been noticed that in T2D patients there is lower methylation of the *PDK4* gene promoter and higher expression than in healthy people. However, it was in healthy people that an increase in transcription was observed after a 4-month increase in physical activity by 5 h/week of Nordic Walking, and no changes were noticed in sick people. This may indicate the inability of patients to respond to such a training program [47].

It was also assessed how DNA methylation in muscles is compared in eldery people who are physically active throughout their lives and in those not active. Hypomethylations in the genes of energy metabolism have been noticed in active against inactive people: related to metabolism of glycogen Glycogen Synthase 2 (*GYG2*) and Glycogen Synthase 1 (*GYS1*), related to the degradation of glycogen Amylase Alpha 2B (*AMY2B*), related to glycolysis ADP Dependent Glucokinase (*ADPGK*), Pyruvate Kinase (*PKM*) and Pyruvate Dehydrogenase Alpha 1 Subunit (*PDHA1*), related to the tricarboxylic acid cycle (TCA) Isocitrate Dehydrogenase (*IDH3A*), or related to ATP/ADP transformations, the mitochondrial SLC25 family of transporters. The content of analyzed protein products of the genes *SLC25A5* and *PDHA1* confirmed the effect of hypomethylation on the increase in the expression of the studied genes. Hypomethylations were observed in 73 genes of mitochondrial metabolism, including the previously described *TFAM*. Interestingly, these 73 genes are regulated by transcription factor 7-like 2 (*TCF7L2*), which is part of the glycogen synthase kinase GSK3/β-Catenin signaling pathway, for which increased expression but not hypomethylation has been reported. Changes in promoter methylation in genes involved in myogenesis and muscle structural dynamics were also significant. The genes Myogenic Differentiation 1 (*MYOD1*), Myosin Light Chain (*MLC*), Ezrin/Radixin/Moesin (*ERM*), dystrophin (*DMD*), Thymosin-4 peptide (*TMSB4*), and genes related to the CDC42 signaling pathway were hypomethylated. Physically active people were also better prepared to manage with oxidative stress through hypomethylation of Microsomal Glutathione S-transferase 1 Gene (*MGST1*), Oxidation Resistance 1 (*OXR1*), Catalase (*CAT*), and Superoxide Dismutase 2 (*SOD2*), which increased gene expression and increased tolerance to oxidative stress. The gene Receptor-type Tyrosine-protein Phosphatase R (*PTPRR*), which inhibits the p38 MAPK pathway, was also hypomethylated, which may contribute to the increased regenerative potential of muscles [23].

The body adapts differently to endurance training versus resistance training, thus one can expect differences in epigenetic regulation. This study based on people in middle-aged with T2D and visceral obesity and both type training 3 days per week, endurance training with progressive-loading exercises and aerobic training cycling by 40–60 min. After endurance training, hypomethylation was noted in Nuclear Respiratory Factor 1 (*NRF1*), Solute Carrier Family 27 Member 4 (*SLC27A4*), Cytochrome P450 (*CYP26C1*), 6-phosphofructo-2-kinase (*PFKFB3*), Histone Deacetylase (*HDAC4*), Glycogen Synthase Kinase 3 Alpha (*GSK3A*), Hexokinase (*HK*), Glucose Transporter Type 4-GLUT4 (*SLC2A4*), and hypermethylation in fatty-acid synthase (*FASN*). However, after resistance training, the genes Glucose transporter type 4-GLUT4 (*SLC2A4*), Acyl-CoA Synthetase Long-chain 1 (*ACSL1*), Low Density Lipoprotein Receptor-related Protein 1 (*LRP1*), Low Density Lipoprotein Receptor-related Protein 10 (LRP10), and Solute Carrier Family 27 member 1 (*SLC27A1*) were hypomethylated [48].

The effect of four weeks of endurance training on epigenetic regulation of muscle adaptation was described in mice, where training induced hypomethylation and increased expression of Insulin-like Growth Factor-binding Protein-4 (*Igfbp4*) and Plexin A2 (*Plxna2*) genes related to muscle growth and differentiation, Docking Protein-7 (Dok7) associated with muscle innervation, and CDP-diacylglycerol Synthase (*Cds2*) associated with angiogenesis [49].

DNA methylation in skeletal muscle cells under the influence of exercise has been widely reported. The adaptation process is very complex and multigene, and the gene products subject to the discussed epigenetic control influence each other, multiplying the importance of this regulation.

### 3.2. In Fat Tissue

The metabolism of adipocytes under the influence of exercise is altered, which are also controlled by DNA methylation processes. Adaptation processes were observed after one-hour session spinning and two one-hour sessions of aerobic per week, and six months of endurance training in a group of middle-aged men who led a sedentary lifestyle. Hypermethylation was observed in adipocytes with a simultaneous decrease in expression in the genes GABA receptors (*GABBR1*), modifying histones Euchromatic Histone Lysine Methyltransferase 1 (*EHMT1*), Euchromatic Histone Lysine Methyltransferase 2 (*EHMT2*) and Histone Deacetylase 4 (*HDAC4*), transcriptional co-repressor the Nuclear Receptor Corepressor 2 (*NCOR2*), and the pathogenesis-related metabolic syndrome and trafficking GLUT4 RalA Binding Protein 1 (*RALBP1*). In addition, hypermethylation of 18 obesity candidate genes was observed, of which two of the Cytoplasmic Polyadenylation Element Binding Protein 4 (*CPEB4*) and Serologically Defined Colon Cancer Antigen 8 (*SDCCAG8*) had reduced expression. Additionally, hypermethylation of 21 out of 39 T2D candidate genes was observed, of which 4, Hematopoietically Expressed Homeobox (*HHEX*), Insulin Like Growth Factor 2 MRNA Binding Protein 2 (*IGF2BP2*), JAZF Zinc Finger 1 (*JAZF1*), and *TCF7L2,* had reduced expression. It is also worth mentioning the T2D candidate gene Potassium Voltage-Gated Channel Subfamily Q Member 1 (*KCNQ1*) which was the most methylated despite the lack of significant differences in expression. This may indicate another stronger control of the expression of this gene [35]. Other studies, on a group of obese adolescents after 6 months of High Intensity Interval Training (HIIT), analyzing changes in the *RALBP1* gene, did not confirm epigenetic modification and expression change [50]. The discrepancies may result from differences in the test group and the type of training.

### 3.3. In Blood Cells

It is interesting to see how exercise affects the body’s inflammatory response, and how DNA methylation plays a role. The gene Apoptosis-associated speck-like Protein Containing a CARD (*ASC*) is important in the inflammatory response. Several studies have demonstrated promoter hypermethylation after moderate exercises in monocytes, granulocytes and PBMC [51,52,53,54]. Moreover, six months of aerobic training revealed hypermethylation of the *ASC* gene promoter [55]. The *PGC-1α* gene was also tested in the blood, where promoter hypomethylation with a decrease in expression was shown in a group of athletic men after acute aerobic exercise (45 min cycling with 75% Wmax and 15 min TT). Additionally, an increase in the expression of inflammatory cytokines *IL-6* and *TNF* as well as methyltransferases *DNMT3A* and *DNMT3B* was noted, but without changes in their methylation [17]. However, a long-term study did not depict a relationship between the level of physical activity and methylation or *PGC-1α* gene expression. The limiting factor for comparing these studies is certainly the age, because in the first case, blood was obtained from adults, and in the second study, the studied group were children under 14 years of age and physical activity was measured be accelerometer [56]. Changes in the expression of IL-6 and TNF under the influence of exercise make them potential measures of physical fitness. Despite considerable involvement after exercise, *IL-6* is not directly under the control of DNA methylation [57,58]. Pro-inflammatory markers that are epigenetically modified are Nuclear Factor Kappa B Subunit 1 (*NFKB1*) and Nuclear Factor Kappa B Subunit 2 (*NFKB2*). After five months of interval aerobic training elderly people, hypermethylation was demonstrated in the promoters of these genes [59]. Biomarker genes for diseases that are subject to this epigenetic control have been identified in blood cells. Twelve weeks HIIT in elderly people contributes to the hypermethylation of the cardiovascular promoter marker of the SHC gene Adaptor Protein 1 (*p66 (Shc)*) [60]. Epigenetic reprogramming was also detected in growth hormone-releasing hormone (*GHRH*) and fibroblast growth factor 1 (*FGF1*) in leukocytes after eight weeks of resistance training (three sets of 8–12 repetitions with 80% maximum load) in young subjects. Both genes were hypomethylated and with increasing transcription of these genes [32].

### 3.4. In Diseased Tissues

The relationship between DNA methylation and neoplastic diseases has been described many times. Much research has been reported to reveal that exercise can affect the methylation of promoters in genes that are important in cancer risk and development. It was observed in PBMC that 16 weeks of aerobic training varied by intensity and duration caused hypomethylation of the suppressor genes Breast Cancer 1 (*BRCA1*), which is normally responsible for DNA break repair, and Polypeptide N-Acetylgalactosaminyltransferase 9 (*GALNT9*), which is involved in uncontrolled polymorphism and metastasis [61]. Another breast cancer suppressor gene found in the blood and undergoing methylation changes after six months of aerobic exercises is Lethal(3)malignant brain tumor-like protein 1 (*L3MBTL1*). High expression of this gene correlated with low-grade tumors and low risk of disease recurrence [62]. The breast cancer suppressor gene of Adenomatosis Polyposis Coli (*APC*) also revealed changes in DNA methylation with exercise. In this case, the physical activity declared in the questionnaire was inversely correlated with promoter hypermethylation in the breast tissue of healthy individuals. The suppressor gene Ras Association Domain Family Member 1 (*RASSF1A*) was also tested, depicting no changes [25]. Based on the methylation of the LINE-1 sequence, it can be seen that exercises reduces the risk of breast cancer by increasing global methylation. In silico studies also noted that LINE-1 methylation status is influenced by long-term exercises, and individual exercise has little effect. This is due to the high stability of LINE-1 methylation over time [20].

The level of physical activity is also important in gastric cancer, where a higher level of methylation of suppressor gene the Calcium Voltage-Gated Channel Auxiliary Subunit Alpha2delta 3 (*CACNA2D3*) has been noticed in less physically active people [63].

Another disease in which a beneficial effect of exercise on development and risk disease via DNA methylation changes of key genes is Nonalcoholic fatty liver disease (*NAFLD*). The expression and methylation of the *MT-ND6* gene is associated with the severity of the *NAFLD* form. Objects determined their level of physical activity by questionnaires. It has been shown that exercise is able to reduce the level of methylation of this gene and therefore exercise can be used to improve the health of patients [64].

Aerobic exercise through methylation was also found to be beneficial for brain development and function. This is due to hypomethylation and an increase in the expression of the brain-derived neurotrophic factor (*BDNF*) gene in the rat’s brain, which improves the functioning and plasticity of the brain [65].

## 4. Expression of Methyltransferases

In the methylation process, it is interesting to control the expression of genes encoding methyltransferases. The enzymes responsible for this genetic modification can be changed by exercise. In the works that were discussed earlier, the researchers sometimes evaluated the expression of methyltransferases. Hunter et al., in the study of *PGC-1α* gene methylation, noticed an increase in *DNMT3A* and *DNMT3B* mRNA in leukocytes after acute exercise (45 min cycling with 75% Wmax and 15 min TT) [17]. In other studies in skeletal muscle, a 50% decrease in the amount of *DNMT3B* mRNA was observed after acute exercise [66]. It was also assessed in PBMC cells how the nuclear concentration of *DNMT3A* and *DNMT3B* changes after acute exercise (120 min running with 60% VO_2max_ and interspersed with sprints at 90% VO_2max_ for the last 30-s of every 10-min). There was no change in the concentration of *DNMT3A*, but there was a significant decrease in the concentration of *DNMT3B*, which may be due to a decrease in expression or nuclear exports. At the same time, researchers noticed an increase in the concentration of *IL-6* in the cytosol. In subsequent stages of the study, they showed that cells stimulated with IL-6 had a decrease in *DNMT3B* nuclear concentration. Researchers also speculated that *DNMT1* expression may be induced by high levels of *IL-6* after exercise, but the study did not verify this [67]. Another decrease in *DNMT3B* mRNA production after 2 weeks endurance exercises was noticed in the hippocampus of rats [68]. The necessity of *DNMT3A* for the right functioning of muscles was demonstrated by Villivalam et al. They detected an increase in *DNMT3A* expression in red muscle cells of mice after endurance exercise. While studying the knockout of the *DNMT3A* gene, they noticed that it contributed to an increase in the expression of the *ALDh1L1* gene, an increase in ROS, mitochondrial dysfunction, and exercise intolerance [69].

DNA methyltransferases can also be controlled by methylation of promoters. The promoter of the *DNMT3B* gene was hypomethylated in skeletal muscle cells in the physically active group compared to the inactive group, which may indicate greater expression and ability to methylate. However, the protein level results showed no difference [23].

The mutual influence of DNA methylation and microRNA (miRNA) on each other also can play a role in the body’s adaptation to exercise. miRNAs are about 22 nucleotides (nt) short non-coding RNA molecules. miRNA binds to messenger RNA (mRNA) leading to silence expression [70]. There are miRNAs that direct on methyltransferase genes to reduce expression. Numerous studies provide evidence of abnormal miRNA expression in cancer which results in decreased expression of DNMT. Certain miRNA can target directly DNMT3A, DNMT3B, and DNMT1 [70,71,72,73]. The expression of miR-29s was found to negatively correlate with DNMT3A and DNMT3B in lung cancer. Increasing miR-29s expression in a lung cancer cell line restored methylation of suppressor genes [74]. On the other hand, miRNA genes can also be methylated. Epigenetic modifications can also influence each other [70,71,75].

Changes in *DNMT* expression due to exercise are one of the mechanisms that contribute to changes in the methylation profile and body function. However, the expression of *DNMT* itself after exercise requires more investigation.

## 5. Active Demethylation

The process of DNA demethylation can be passive through the loss of 5-mC in subsequent replications. The active process takes place with the use of enzymes in especially from the TET family, but also DNMT.

DNMT3A and DNMT3B methyltransferases have demethylase activity. It is a process independent of other active demethylation processes, but the exact mechanism requires further research [76].

**Figure 4:**
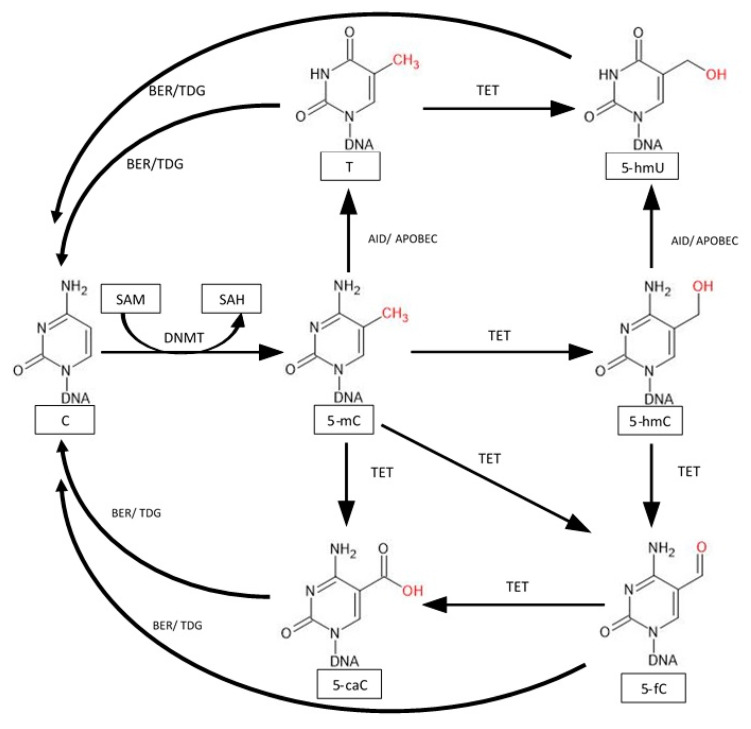
Modification of 5-methylcytosine leads to the restoration of unmethylated cytosine. 5-mC is oxidized by TET protein to 5-hmC, 5-formylcytosine (5-fc), and 5-carboxylcytosine (5caC). Thymine (T) can also be oxidized by TET to 5-Hydroxyuracil (5-hmU). Deamination of 5-mC and 5-hmC by an enzyme complex Activation-induced deaminase/apolipoprotein B mRNA-editing catalytic polypeptide-like (ADI/APOBEC) leads to the formation of thymine and 5-hmU. The final step in restoring cytosine occurs with Base Excision Repair (BER) and thymine DNA glycosylase (TDG), which modify 5-fc, 5-caC, 5-hmU, and T [76,77,78,79,80].

One of the main processes of active demethylation begins with the conversion of 5-mC to 5-hmC by proteins of the TET family. The TET1 protein has a CXXC domain thanks to which it binds to methylated and unmethylated CpG sequences and transforms 5-mC into 5-hmC and further removes methylation markers and reactivating silenced genes [77]. The TET3 protein also has this domain with the difference that it binds to DNA independent of the presence of guanine. The TET2 protein has lost the sequence containing this domain and is dependent on the CXXC Finger Protein 4 (IDAX) protein which has this domain. During the conversion of 5-mC to 5-hmC, 2-oxoglutarate (2-OG) is oxidized to succinate and CO_2_. TET proteins can also convert 5-hmC to 5-fC and 5-caC. However, TET proteins cannot completely demethylate, because they cannot convert the final 5-caC product. The last step takes place with the participation of other proteins, such as DNMT or TDG (Figure 4) [78].

Exercise and aging can also affect TET protein expression. TET protein expression decreases over time, and exercise is able to stop these changes. An effect was seen in the hippocampus of old rats, but not in the hypothalamus [79]. A decrease in TET1 expression under the influence of exercise was observed in arterial myocytes of rats [80]. The involvement of TET family proteins in exercise-induced demethylation is a little explored topic, but several studies show that there is a modification of the expression of these genes.

## 6. Summary

Accurate determination of the effects of DNA methylation on individual genes and globally is problematic in some cases. Comparing global methylation across studies presents a problem due to different techniques for determining this level. Some of them appear to be less precise, such as the LUMA test. Moreover, the use of a different matrix makes it possible to detect or omit sites of altered methylation that were recognized in other research protocols [39]. A direct example of potential mistakes was presented by Lindholm et al., who observed increased methylation after exercising two CpG sites, as in the work of Nitert et al. The difference was in the location of the modified nucleotides, because the first noticed this change in the gene’s body, and the second in the promoter region, which may already affect transcriptions [33].

Despite these difficulties, DNA methylation testing depicts how extensive changes exercise makes to the body’s functioning. Already single exercises can affect the methylation of many genes, which leads to modifications of the expression genetic material and reveals the complexity of the body’s adaptive and regulatory mechanisms after exercise. Physical activity over a longer period of time has a greater effect on DNA methylation and body adaptation. Stronger changes in this epigenetic modification were noticed after regular exercises and training periods, which can be explained by epigenetic memory. An interesting but time-consuming topic would be to analyze how different training and rest periods affect DNA methylation, for example, in the training cycle of professional athletes.

A better understanding of the effects of exercise on DNA methylation will help understand their impact on pathomechanisms in metabolic diseases, including T2D. Thanks to work such as Rowlands et al. [48], it is possible to trace the relationships between DNA methylation of individual genes and metabolic pathways. Research work allows us to supplement the knowledge about the influence of exercise on energy metabolism.

Research on the effects of supplementation and nutrition on the impact of exercise on the level of DNA methylation may be of interest. Some nutritional practices in laboratory studies show inconclusive results, such as fatty acids (FA) for reducing inflammation [17]. Looking at the issue in terms of gene methylation can help accurately assess the effects of supplements or compounds in food on body function.
